# Reduced *SKP1* and *CUL1* expression underlies increases in Cyclin E1 and chromosome instability in cellular precursors of high-grade serous ovarian cancer

**DOI:** 10.1038/s41416-021-01317-w

**Published:** 2021-03-17

**Authors:** Chloe Camille Lepage, Michaela Cora Lynn Palmer, Ally Catherina Farrell, Nicole Marie Neudorf, Zelda Lichtensztejn, Mark William Nachtigal, Kirk James McManus

**Affiliations:** 1 Department of Biochemistry & Medical Genetics, University of Manitoba, Winnipeg, Manitoba Canada; 2 Research Institute in Oncology & Hematology, CancerCare Manitoba, Winnipeg, Manitoba Canada; 3 Department of Obstetrics, Gynecology & Reproductive Sciences, University of Manitoba, Winnipeg, Manitoba Canada

**Keywords:** Ovarian cancer, Ovarian cancer, Genetics research

## Abstract

**Background:**

High-grade serous ovarian cancer (HGSOC) is the most common and lethal ovarian cancer histotype. Chromosome instability (CIN, an increased rate of chromosome gains and losses) is believed to play a fundamental role in the development and evolution of HGSOC. Importantly, overexpression of Cyclin E1 protein induces CIN, and genomic amplification of *CCNE1* contributes to HGSOC pathogenesis in ~20% of patients. Cyclin E1 levels are normally regulated in a cell cycle-dependent manner by the SCF (SKP1–CUL1–FBOX) complex, an E3 ubiquitin ligase that includes the proteins SKP1 and CUL1. Conceptually, diminished *SKP1* or *CUL1* expression is predicted to underlie increases in Cyclin E1 levels and induce CIN.

**Methods:**

This study employs fallopian tube secretory epithelial cell models to evaluate the impact diminished *SKP1* or *CUL1* expression has on Cyclin E1 and CIN in both short-term (siRNA) and long-term (CRISPR/Cas9) studies.

**Results:**

Single-cell quantitative imaging microscopy approaches revealed changes in CIN-associated phenotypes and chromosome numbers and increased Cyclin E1 in response to diminished *SKP1* or *CUL1* expression.

**Conclusions:**

These data identify *SKP1* and *CUL1* as novel CIN genes in HGSOC precursor cells that may drive early aetiological events contributing to HGSOC development.

## Background

High-grade serous ovarian cancer (HGSOC) is the most common histotype of epithelial ovarian cancer, accounting for ~70% of all diagnoses.^1,2^ Owing to the lack of early detection methods, most women are diagnosed at late stages (~51% and ~29% at stages III and IV, respectively,^3^) and are typically treated with surgical debulking and neoadjuvant or adjuvant chemotherapy. Unfortunately, ~90% of patients ultimately succumb to the drug-resistant disease, and thus greater insight into the aetiological origins is required to improve early detection and positively impact the outcomes of women living with HGSOC. HGSOC is commonly referred to as a ‘genetically unstable’ tumour type with a complex genome^4–9^ consisting of a large number of low-frequency gene copy number alterations and structural changes;^6,10,11^ however, the molecular determinants giving rise to this genome instability still remain largely unknown.

Genome instability is an enabling hallmark in many cancer types^12^ that is classically categorised into three main forms: microsatellite instability, CpG island methylator phenotype and chromosome instability (CIN). Whilst microsatellite instability and CpG island methylator phenotype tend to be less prevalent in cancers, CIN is a predominant form of genome instability defined by an increase in the rate at which whole chromosomes or large parts thereof are gained or lost.^13^ CIN induces ongoing changes in chromosome complements that adversely impact large cohorts of genes (e.g., oncogenes, tumour suppressor, DNA repair and apoptotic genes) and is a driver of cell-to-cell heterogeneity. As a direct consequence, CIN is an enabling phenotype in many cancer types that is associated with cancer initiation, intratumoural heterogeneity, metastasis, drug resistance and poor patient outcomes.^14–18^ The prevalence and dynamics of CIN were only recently correlated with drug resistance and disease recurrence in HGSOC,^19^ and Nelson et al.^20^ demonstrated highly rearranged chromosomes and chromosome segregation defects within isolated HGSOC cultures. Recent genetic and molecular data suggest that CIN may also harbour a role in early disease development.^21,22^ Indeed, genomic amplification of the Cyclin E1 gene (*CCNE1*) and subsequent overexpression of Cyclin E1 occur in both precursor lesions and HGSOCs.^21,22^ Cyclin E1 is a prototypic cell cycle-regulated protein, whose expression is temporally regulated by the ubiquitin–proteasome pathway, specifically by the SKP1–CUL1–F-box protein (SCF) complex.^23^ Normally, Cyclin E1 orchestrates the G1- to S-phase transition,^24^ but it also regulates DNA replication and centrosome biology.^25^ Importantly, high Cyclin E1 expression has been determined to induce CIN in various cancer contexts.^21,26,27^ Notably, genomic *CCNE1* amplification occurs in 10–20% of HGSOCs;^28,29^ however, high Cyclin E1 protein expression occurs in ~30–50% of HGSOCs,^28,30,31^ suggesting additional mechanisms exist to account for high Cyclin E1 expression, which likely also drives HGSOC pathogenesis.

To accurately assess CIN and identify CIN genes mandates the use of techniques that are capable of analysing either (1) continuously growing cultures derived from a single cell over time (temporal analysis) or (2) single-cell approaches capable of quantifying the result of CIN (i.e., cell-to-cell heterogeneity) within a given population at a given time point (endpoint analysis).^32^ Over the past decade, pioneering efforts coupling quantitative imaging microscopy (QuantIM) with endpoint analyses have enabled the assessment of CIN at the single-cell level.^19,33–35^ In this regard, QuantIM is a rapid approach capable of capturing the cell-to-cell variation in aberrant phenotypes, including changes in nuclear areas and micronucleus (MN, extranuclear body found outside the primary nucleus) formation. Conceptually, changes in nuclear areas are typically associated with large-scale changes in DNA content or chromosome numbers (i.e., ploidy),^36–39^ while micronuclei typically arise due to chromosome missegregation events and are a hallmark of CIN.^40–42^ Thus, identifying the molecular determinants of CIN is not only essential to gain novel insight into HGSOC pathogenesis, but may uncover novel disease biomarkers or reveal genetic susceptibilities that can be therapeutically exploited (reviewed in ref. ^17^).

The ubiquitin–proteasome pathway represents the major mechanism mediating targeted intracellular protein turnover.^43^ This pathway involves the conjugation of ubiquitin to protein substrates within the cell, which targets them for proteolytic degradation by the 26S proteasome.^38^ The SCF complex is an E3 ubiquitin ligase that is involved in the degradation of Cyclin E1.^23^ The SCF complex is composed of four protein subunits, three of which are invariable core components (RING box protein 1 (RBX1), S-phase kinase-associated protein 1 (SKP1) and cullin 1 (CUL1)) and a variable F-box protein that confers substrate specificity.^23^ A recent study showed that the assembly of specific SCF complexes (i.e., containing one of the 68 different F-box proteins) is dictated by substrate abundance, and thus demonstrates that the cellular repertoire of SCF complexes is dynamic.^44^ Within an assembled SCF complex, SKP1 serves as an adapter that binds an F-box protein and associated substrate;^45^ RBX1 recruits an E2 ubiquitin-conjugating enzyme from which ubiquitin will ultimately be transferred to the target substrate,^46^ and CUL1 serves as a scaffold linking RBX1 to SKP1 that, upon activation, undergoes a conformational change to increase spatial proximity between these two components.^47^ Recently, Thompson et al. showed that reduced *SKP1* expression correlated with increased Cyclin E1, CIN and endoreduplication in a colorectal cancer context.^39^ Moreover, genetic rescue experiments were conducted that demonstrated reduced CIN phenotypes when both *SKP1* and *CCNE1* were co-silenced. These data support the possibility that aberrant Cyclin E1 turnover caused by a disruption in SCF complex activity drives CIN. We hypothesise that altered expression of SCF complex core proteins plays a prominent role in HGSOC pathogenesis by enhancing CIN. The current study is focused on two core components of the SCF complex, SKP1 and CUL1, and their potential roles in regulating chromosome stability. Using siRNA and/or CRISPR/Cas9 approaches, we determined that reduced *SKP1* or *CUL1* expression induced CIN in two fallopian tube secretory epithelial (FT) cell models, a cellular precursor of HGSOC.^48,49^ Collectively, our data show that diminished SKP1 or CUL1 expression induces CIN, which is consistent with potential pathogenic roles in HGSOC.

## Methods

### Cell culture

FT194 (human TERT, SV40 large T antigen) and FT246 (human TERT, *TP53*-shRNA, human CDK4.R24C overexpression) fallopian tube secretory epithelial cell lines were generously provided by Dr. R. Drapkin (University of Pennsylvania, USA). Cells were cultured in DMEM/F12 medium (Gibco) supplemented with 2% Ultroser G serum substitute (Pall Corporation, Pall France) and maintained at 37 °C in a humidified incubator (5% CO_2_).

### Genomic alterations

Genomic alteration data generated by The Cancer Genome Atlas (TCGA, https://portal.gdc.cancer.gov/)^50^ were used in all analyses. Publicly available data were extracted from 12 cancer types using web-based analysis and visualisation tools located at cBioPortal (www.cbioportal.org).^51^ User-defined onco-query commands (HETLOSS, HOMDEL) were used to extract copy number variations for each query gene. Putative copy number alterations from cBioPortal are estimates generated from patient data using GISTIC,^52^ where sample-specific thresholds are applied to identify regions possessing deep deletions, shallow deletions or neutral copy numbers as detailed elsewhere.^52,53^ Accordingly, low-level thresholds are determined on an individual sample basis and are based on the minimum median arm-level deletion copy number identified for a given sample.

### Gene silencing, western blots, RNA isolation and quantitative polymerase chain reaction

Transient gene silencing was performed by transfecting *SKP1* or *CUL1* siRNA duplexes into cells using RNAiMax (Life Technologies, Burlington, Ontario, Canada), as detailed elsewhere.^54,55^ Briefly, cells were seeded, permitted to attach for 24 h, and four ON-TARGETplus (GE Dharmacon, Lafayette, CO, USA) duplexes targeting distinct coding sequence regions within each gene were used either individually or as a Pool. A non-targeting siRNA served as negative control (siControl). Gene silencing was assessed 4 or 6 days post transfection by western blots, using the antibodies and dilutions indicated in Supplementary Table SI. Semi-quantitative western blotting was performed to determine silencing efficiencies using ImageJ, where protein abundance was first normalised to the corresponding loading control (α-tubulin or cyclophilin B) and is presented relative to the siControl (set to 1.00).

To identify whether changes in *CCNE1* RNA following *SKP1* or *CUL1* silencing, total RNA was harvested 4 days (FT194) or 6 days (FT246) post transfection. Generation of cDNA and quantitative RT-PCR were conducted as previously described^56^ with the exception that Superscript III was used for the first-strand synthesis and the cycle parameters were 40 cycles 15 s at 94 °C, 20 s at 60 °C.

### Single-cell quantitative imaging microscopy

Cells were seeded into 96-well optical bottom plates and silenced. At 4 days (FT194) or 6 days (FT246) post transfection, cells were fixed (4% paraformaldehyde) and nuclei were counterstained (Hoechst 33342). A 3 × 3 matrix of non-overlapping 2D images (i.e., nine images total) was acquired from each well using a Cytation 3 Cell Imaging Multi-Mode Reader (BioTek, Winooski, VT, USA) equipped with a 16-bit, greyscale, charge-coupled device camera and a ×20 (0.45 numerical aperture) lens. All image acquisition and analysis settings were adjusted using Gen5 Software (BioTek), with representative images exported into Photoshop CS6 (Adobe) for figure assembly. Nuclear areas were determined as detailed elsewhere.^34,36,38,39^ Briefly, the primary mask function of Gen5 software was employed to automatically detect interphase Hoechst-labelled nuclei above a minimum signal intensity threshold, which was optimised for each experiment and held constant between all experimental conditions. Inclusion filters were employed to detect objects of a pre-defined diameter (10 μm ≤ × ≥100 μm), while an *xy* boundary exclusion filter was employed to remove partial nuclei located along the image periphery. Nuclear areas were automatically calculated, and data were exported into Prism v8 (GraphPad), where descriptive statistics and two-sample Kolmogorov–Smirnov (K–S) tests were performed and graphs were generated.

Images were assessed for MN formation as detailed elsewhere.^35–39^ In short, Gen5 (BioTek) image analysis software was employed to automatically detect micronuclei, which were operationally defined as small, extra-nuclear, Hoechst-stained bodies (≤1/3 the size of the primary nucleus) exhibiting no visible attachments with the primary nucleus. A primary mask was applied to detect primary interphase nuclei as described above, while a secondary mask was applied with a defined ring width (15 μm) to approximate the cell boundary. Next, Spot Detection was employed to identify micronuclei (i.e., spots) located outside the primary nucleus, but within the cell boundary. A size-inclusion filter was also applied to restrict MN identification to Hoechst-stained bodies between 1 and 6 μm in diameter, while a maximum intensity threshold was used to exclude brightly stained apoptotic bodies and mitotic chromosomes. Finally, an *xy* boundary exclusion filter was applied to eliminate partial nuclei located along the image periphery. The total number of micronuclei in each well was determined and normalised to the total number of nuclei, with wells containing fewer than 40 nuclei excluded from subsequent analyses. MN formation data were imported into Prism v8 where descriptive statistics and Mann–Whitney (M–W) statistical tests were performed.

### Mitotic chromosome spreads, SKY and chromosome enumeration

Mitotic chromosome spreads were generated, as detailed previously,^55^ with SKY being performed on FT194 and FT246 cell lines as detailed elsewhere.^54^ Mitotic chromosome enumeration was performed by seeding 20,000 FT194 or FT246 cells into six-well plates, with siRNAs targeting *SKP1*, *CUL1* or siControl transfected into cells as indicated above. Mitotic chromosome spreads were generated and a minimum of 100 spreads/condition were imaged and manually enumerated using FIJI software. Spreads harbouring modal chromosome numbers were classified as normal, whereas those harbouring non-modal numbers were considered aberrant and classified into one of four categories: (1) small-scale losses (<10 chromosomes lost), (2) small-scale gains (<10 chromosomes gained), large-scale losses (≥10 chromosomes lost) and large-scale gains (≥10 chromosomes gained). The threshold number of ten chromosomes was determined based on the observation that chromosome numbers generally clustered either in the near-diploid region (i.e., 46 ± 5 chromosomes) or in the near-tetraploid region (i.e., 92 ± 5 chromosomes), with very few spreads containing an intermediate number of chromosomes. Thus, a threshold of 10 chromosomes were chosen as an intermediate threshold value to distinguish between these two populations (i.e., small-scale vs. large-scale chromosome gains/losses). A mitotic chromosome spread enumeration was performed twice/condition (*n* = 2) for each cell line, with all data imported into Prism v8 where descriptive statistics, two-sample K–S tests and graphs were generated.

### Phenotypic rescue

Individual or co-silencing experiments targeting *SKP1*, *CUL1* or *CCNE1* and siControl were performed using the siRNA-based approach detailed above (see the section ‘Gene silencing, western blots, RNA isolation and quantitative polymerase chain reaction’). Briefly, FT246 cells were transfected with individual or combinations of equimolar siRNA pools, permitted to grow for 6 days at which point semi-quantitative western blots and QuantIM (nuclear areas and MN formation) were performed as above. Two-sample K–S tests were used to identify significant differences in cumulative nuclear area distribution frequencies, while ANOVA and Tukey multi-comparison post tests were used to identify changes in MN formation.

### CRISPR/Cas9 editing of *SKP1*

CRISPR/Cas9 was employed to generate *SKP1* heterozygous knockout (*SKP1*^+/−^) FT246 cells using a two-step approach as indicated by the manufacturer (Sigma, Aldrich, Oakville, Canada). Briefly, FT246 cells were transduced with lentiviruses containing a synthetic guide RNA (sgRNA) and blue fluorescent protein (BFP) expression cassette. Cells were transduced with two distinct *SKP1*-targeting sgRNAs or a non-targeting (NT-control) sgRNA, and fluorescence-activated cell sorting was used to isolate transduced cells. Cells were subsequently transfected with a Cas9 and green fluorescent protein (GFP) expression plasmid and fluorescence-activated cell sorting was used to isolate transfected (BFP- and GFP-positive) cells. Individual CRISPR-edited clones were isolated as single cells using serial dilutions from the bulk population, clonally expanded and screened for diminished *SKP1* expression via western blot. Putative CRISPR/Cas9-edited clones were confirmed by DNA sequencing (Genome Quebec, Montreal, Quebec, Canada) and allele-specific edits were determined through comparisons with the reference sequence.

## Results

### Determining the prevalence of diminished *SKP1* or *CUL1* expression in HGSOC

**Fig. 1:**
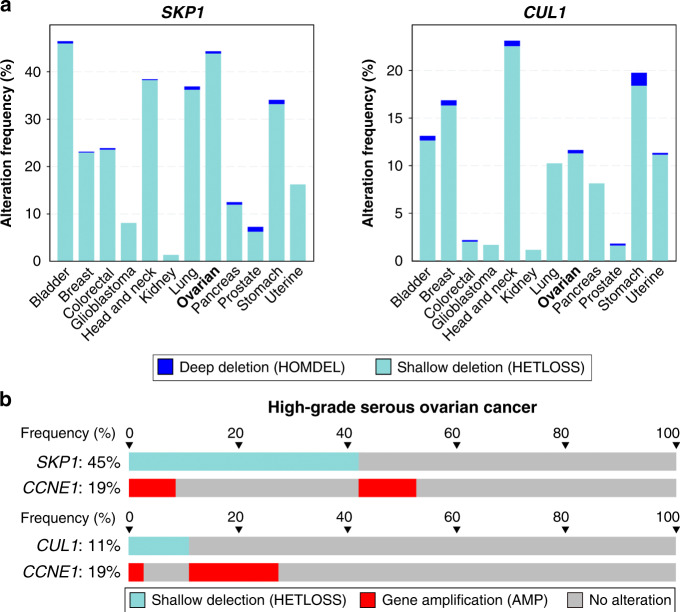
*SKP1* and *CUL1* are frequently lost in numerous cancer types, including HGSOC. **a** Column graphs depicting the frequency of *SKP1* (left) or *CUL1* (right) deep (homozygous) and shallow (heterozygous) deletions in 12 common cancer types.^50,51^ **b** Bar graphs comparing heterozygous loss of either *SKP1* (top) or *CUL1* (bottom) with *CCNE1* amplification identify instances of mutual exclusivity within HGSOC patient samples (398 total cases). Vertical alignments identify samples from the same patient.

To begin to assess the potential relevance of *SKP1* or *CUL1* alterations in cancer, the frequency of *SKP1* or *CUL1* copy number losses was assessed using TCGA data obtained for 12 common cancer types (Fig. 1a).^50,51^ In general, deep (i.e., homozygous) deletions are rare for both genes and typically range from 0 to 1%. Alternatively, shallow (i.e., heterozygous) deletions are more frequent for both genes and are present in all 12 cancer types. With respect to HGSOC, heterozygous loss of *SKP1* or *CUL1* occurs in ~45% (143/316) and ~13% (40/316) of cases, respectively (Fig. 1b).^10^ Only 7% of patients exhibit heterozygous loss of both *SKP1* and *CUL1*, while cumulative heterozygous loss occurs in 44.0% (139/316) of HGSOC patients. Of the 143 patient tumours harbouring *SKP1* shallow deletions, ~78% (111/143) occur independent of *CCNE1* amplification, while ~22% (32/143) co-occur with *CCNE1* gene amplification (Fig. 1b). A single HGSOC patient sample harbouring a *SKP1* deep deletion was identified, and this alteration was mutually exclusive with *CCNE1* amplification. Similarly, of the 20 patient samples harbouring *CUL1* shallow deletions, ~85% (34/40) are mutually exclusive, while ~15% (6/40) co-occur with *CCNE1* amplification; no HGSOC patient samples were identified with homozygous loss of *CUL1*. Thus, *SKP1* and *CUL1* loss have a tendency towards mutual exclusivity with *CCNE1* amplification, which supports the possibility that their aberrant expression underlies increases in Cyclin E1 protein levels. Collectively, these data suggest that while complete loss of *SKP1* or *CUL1* expression may not be compatible with cell viability (i.e., essential genes), reduced *SKP1* or *CUL1* expression may have potential roles in cancer initiation.

### Identifying a clinically relevant cellular precursor model for HGSOC in which to evaluate CIN

Recent molecular, genetic and clinical evidence strongly suggests that HGSOCs arise from secretory epithelial cells within the distal fallopian tube.^49,57–61^ As such, immortalised FT cell lines^62^ are being increasingly employed to model the early genetic events suspected to underlie cellular transformation and HGSOC development.^7,21,48,63,64^ FT194 and FT246 are two FT cell lines currently employed in HGSOC research contexts,^63,65–70^ and to determine whether these models are suitable for CIN studies (i.e., karyotypically stable), their baseline karyotypes were assessed in mitotic chromosome spreads using spectral karyotyping (SKY). SKY revealed that FT194 cells contain 46 chromosomes (Supplementary Fig. S1A), with a three-way chromosomal rearrangement involving chromosomes 5, 14 and 20 (46,XX,t(5;14;20)(p1?5;q2?4;p1?3)), while FT246 cells appear to have two predominant sub-populations harbouring a derivative chromosome 7 containing a fragment from chromosome 3, along with some subclone-specific rearrangements (Supplementary Fig. S1B). Accordingly, SKY determined that FT246 subclone 1 (46,XX,der(7)t(3;7),−10,−10,+der(10)t(10;X)x2,del(X)(q?)) and 2 (45,XX,der(4)t(1;4),der(7)t(3;7),del(10)(p11.2),−13,−21,+mar) have 46 and 45 chromosomes, respectively. Importantly, continual passaging over ~3 months established that each line maintained these karyotypes and are therefore karyotypically stable, which is essential for CIN-based studies. Accordingly, these data identify FT194 and FT246 as ideal cell models in which to study CIN.

### *SKP1* or *CUL1* silencing induces increases in Cyclin E1 levels in FT cells

**Fig. 2:**
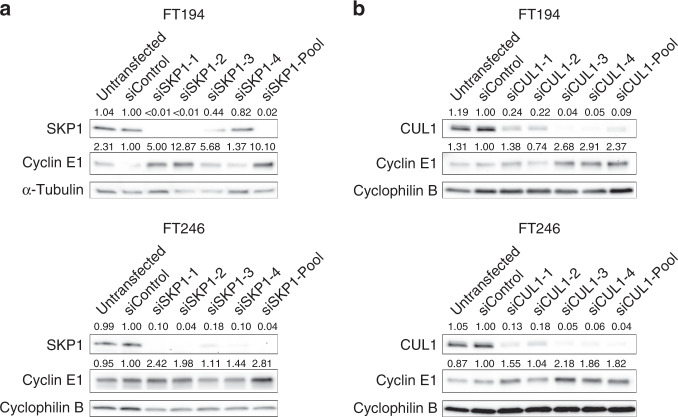
*SKP1* or *CUL1* silencing is associated with increases in Cyclin E1 abundance. **a** Semi-quantitative western blots depicting the *SKP1* silencing efficiency of the individual (siSKP1–1, siSKP1–2, siSKP1–3 and siSKP1–4) and four pooled (siSKP1-Pool) siRNAs within FT194 (top) and FT246 (bottom) cell lines. Note the increases in Cyclin E1 levels within the *SKP1* silenced cell populations. Fold changes in SKP1 or Cyclin E1 expression relative to siControl are indicated, using cyclophilin B or α-tubulin as loading controls. *n* = 3. **b** Semi-quantitative western blots depicting the *CUL1* silencing efficiency of the individual (siCUL1–1, siCUL1–2, siCUL1–3 and siCUL1–4) and four pooled (siCUL1-Pool) siRNAs within FT194 (top) and FT246 (bottom) cell lines. Note the increases in Cyclin E1 levels within the *CUL1* silenced cell populations. Fold changes in CUL1 or Cyclin E1 expression relative to siControl are indicated, using cyclophilin B as a loading control. *n* = 3.

The role of the SCF complex in regulating Cyclin E1 levels suggests that altered expression or function of SCF complex components, like *SKP1* or *CUL1*, may lead to altered target protein turnover and CIN.^39^ However, prior to testing this hypothesis, the silencing efficiency of four individual siRNAs or a pooled siRNA (comprising equal molar amounts of the four individual siRNAs) targeting *SKP1* or *CUL1* was evaluated by semi-quantitative western blot analyses. For each gene, two of the four siRNA duplexes consistently resulted in efficient silencing (≤10% of endogenous protein expression) in both cell lines, namely siSKP1–1 and siSKP1–2 (Fig. 2a) or siCUL1–3 and siCUL1–4 (Fig. 2b). Accordingly, these siRNAs along with the pooled siRNA were employed in all subsequent experiments. As expected, reduced *SKP1* or *CUL1* expression correlated with increased Cyclin E1 levels with the conditions inducing the greatest decreases in *SKP1* or *CUL1* expression corresponding with the largest increases in Cyclin E1 levels (Fig. 2 and Supplementary Fig. S2). For example, siSKP1-Pool silencing in FT194 and FT246 cells resulted in 10.1-fold and 2.8-fold increases in Cyclin E1 levels, respectively, while siCUL1-Pool silencing resulted in 2.4-fold and 1.8-fold increases in Cyclin E1 levels within the two cell lines, respectively. Analysis of *SKP1* and *CUL1* transcript levels by quantitative RT-PCR showed a significant decrease in *SKP1* in FT194 cells, but a modest increase (1.3-fold) in FT246 cells (Supplementary Fig. S3). No differences were observed for *CUL1*. Thus, diminished *SKP1* or *CUL1* expression induced increased Cyclin E1 levels, which is predicted to contribute to CIN in FT cells.

### Transient *SKP1* or *CUL1* silencing induces changes in nuclear areas and increases in MN formation in FT cells

**Fig. 3:**
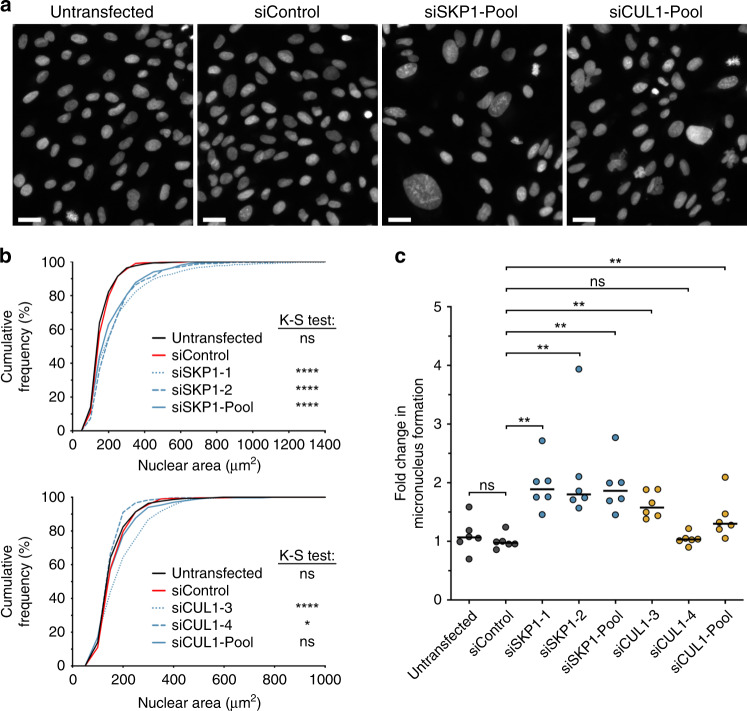
*SKP1* or *CUL1* silencing induces increases in CIN-associated phenotypes in FT194 cells. **a** Representative images of Hoechst-counterstained nuclei showing qualitative changes in nuclear areas following *SKP1* or *CUL1* silencing. Scale bar represents 30 μm. **b** Nuclear area cumulative frequency distributions following *SKP1* (top) or CUL1 (bottom) silencing relative to siControl. K–S tests reveal statistically significant changes (i.e., rightward shift) in nuclear area cumulative frequency distributions relative to siControl (ns not significant, *P value <0.05; ****P value < 0.0001). *n* = 3; 300 nuclei analysed/condition. **c** Dot plot depicting mean fold changes in MN formation following *SKP1* or *CUL1* silencing, relative to siControl. Black lines identify the median of six replicate wells. Statistical significance is indicated (Mann–Whitney [M–W] test; ns not significant; **P value < 0.01). *n* = 3; *n* = 6.

**Fig. 4:**
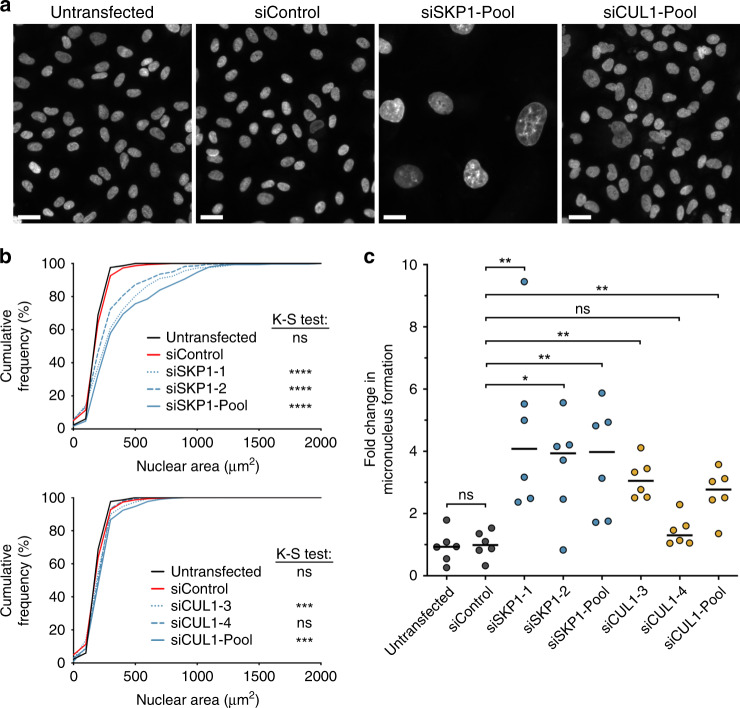
*SKP1* or *CUL1* silencing induces increases in CIN-associated phenotypes in FT246 cells. **a** Representative images of Hoechst-counterstained nuclei showing qualitative changes in nuclear areas following *SKP1* or *CUL1* silencing. Scale bar represents 30 μm. **b** Cumulative nuclear area frequency distributions following *SKP1* (top) or *CUL1* (bottom) silencing relative to siControl. K–S tests reveal statistically significant changes (i.e., rightward shift) in nuclear area distributions relative to siControl (ns not significant; ***P value < 0.001; ****P value < 0.0001). *n* = 3; 300 nuclei analysed/condition. **c** Dot plot depicting mean fold changes in MN formation following *SKP1* or *CUL1* silencing relative to siControl. Black lines identify the median of six replicate wells. Statistical significance is indicated (Mann–Whitney [M–W] test; ns not significant; *P value < 0.05; **P value < 0.01). *n* = 3; *n* = 6. Statistical significance is indicated (ns not significant; *P value < 0.05; **P value < 0.01).

To determine the impact diminished *SKP1* or *CUL1* expression has on CIN, we employed QuantIM to assess changes in nuclear areas and MN formation following *SKP1* or *CUL1* silencing in FT194 and FT246 cells. As predicted, reduced *SKP1* or *CUL1* expression in FT194 resulted in visual increases in nuclear area heterogeneity (Fig. 3a) that corresponded with statistically significant changes (i.e., rightward or leftward shifts) in cumulative distribution frequencies (Fig. 3b) relative to siControl (see Supplementary Tables SII–SIII). Similarly, reduced expression of SKP1 or CUL1 generally correlated with significant increases in MN formation relative to siControl, with the exception of siCUL1–4 (Fig. 3c). Furthermore, *SKP1* and *CUL1* silencing in FT246 (Fig. 4) also corresponded with visual increases in nuclear areas, significant increases in cumulative nuclear area distribution frequencies (Supplementary Table SIV) and significant increases in MN formation (Supplementary Table SV), again with the exception of siCUL1–4. Interestingly, *SKP1* silencing in both FT194 and FT246 cells was generally associated with larger changes in nuclear area distributions and increases in MN formation than that induced following *CUL1* silencing. Collectively, these data suggest that *SKP1* and *CUL1* are putative CIN genes in two FT cellular contexts.

### *CCNE1* co-silencing rescues the CIN phenotypes associated with reduced *SKP1* or *CUL1* expression

**Fig. 5:**
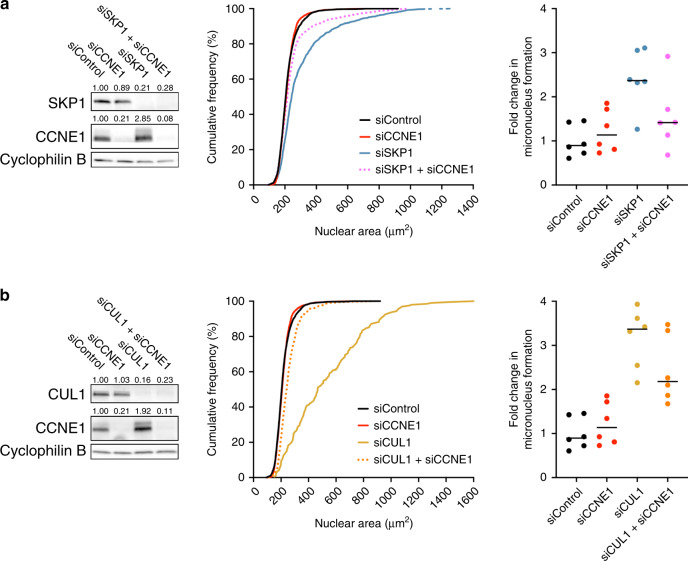
Co-silencing Cyclin E1 (CCNE1) rescues the CIN phenotypes. **a** Semi-quantitative western blot (left) presenting the silencing efficiencies following individual or co-silencing of siControl, siSKP1, siCCNE1 or siSKP1 + siCCNE1; cyclophilin B serves as a loading control. Numerical values above each lane identify the residual protein levels relative to siControl (1.00). Cumulative nuclear area distribution frequency graph (middle) shows that co-silencing siSKP1 + siCCNE1 (dotted magenta line) induces a significant reduction in nuclear area distribution (KS test, *P* value <0.0001) relative to siSKP1 alone (blue line). Dot plot (right) reveals a decrease in MN formation following siSKP1 + siCCNE1 relative to siSKP1 alone (see Supplementary Table SVI). Horizontal black bars are the median of each condition. **b** Western blot (left) depicting the silencing efficiencies of individual and combined siRNA treatments with the numerical values indicating residual protein levels. Cumulative nuclear area distribution frequency graph (middle) reveals co-silencing (siCUL1 + siCCNE1) produces a significant (*P* value < 0.0001) phenotypic rescue relative to siCUL1 alone. Dot plot (right) also shows a decrease in MN formation following co-silencing relative to siCUL1 alone (see Supplementary Table SVII).

While *CCNE1* is amplified in ~10–20% of HGSOC cases^28,29^ and ectopic overexpression induces CIN in animal models,^21^ ~30–50% of all HGSOC cases exhibit increased levels of Cyclin E1 protein,^28,30,31^ suggesting additional mechanisms beyond genomic amplification exist to account for the elevated levels and CIN. Since SKP1 and CUL1 (i.e., SCF complex) normally regulate Cyclin E1 levels, we sought to gain mechanistic insight by determining whether aberrant Cyclin E1 turnover was responsible for the CIN phenotypes observed following *SKP1* or *CUL1* silencing. To test this possibility, genetic rescue experiments were performed to determine if co-silencing *CCNE1* would rescue the aberrant CIN phenotypes observed when *SKP1* or *CUL1* are silenced alone. We first confirmed our initial observation that *SKP1* and *CUL1* silencing corresponded with increases in Cyclin E1 abundance and further established our ability to co-silence *SKP1* + *CCNE1* and *CUL1* + *CCNE1* (Fig. 5a, b, left panels). As above, QuantIM identified statistically significant increases in cumulative nuclear area frequency distributions following silencing of either *SKP1* or *CUL1*; however, these large increases were dramatically reduced following co-silencing of *CCNE1* (Fig. 5a, b, middle panels). Similarly, *SKP1* or *CUL1* silencing induced significant increases in MN formation, while *CCNE1* co-silencing reduced MN formation (Fig. 5a, b, right panels; Supplementary Tables SVI and SVII). Collectively, these data demonstrate that increases in Cyclin E1 protein levels contribute, at least in part, to the CIN phenotypes associated with reduced *SKP1* or *CUL1* expression. Furthermore, as complete genetic rescues did not occur despite efficient CCNE1 silencing, these findings suggest additional SCF targets may also contribute to the CIN phenotypes observed following *SKP1* or *CUL1* silencing. Nonetheless, these data show that diminished *SKP1* or *CUL1* expression (i.e., aberrant SCF complex function) corresponds with increases in Cyclin E1 levels that collectively induce CIN, and are therefore consistent with reduced *SKP1* or *CUL1* expression being novel pathogenic drivers of HGSOC that are independent of genomic *CCNE1* amplification.

### *SKP1* or *CUL1* silencing induces changes in chromosome numbers in FT cells

**Fig. 6:**
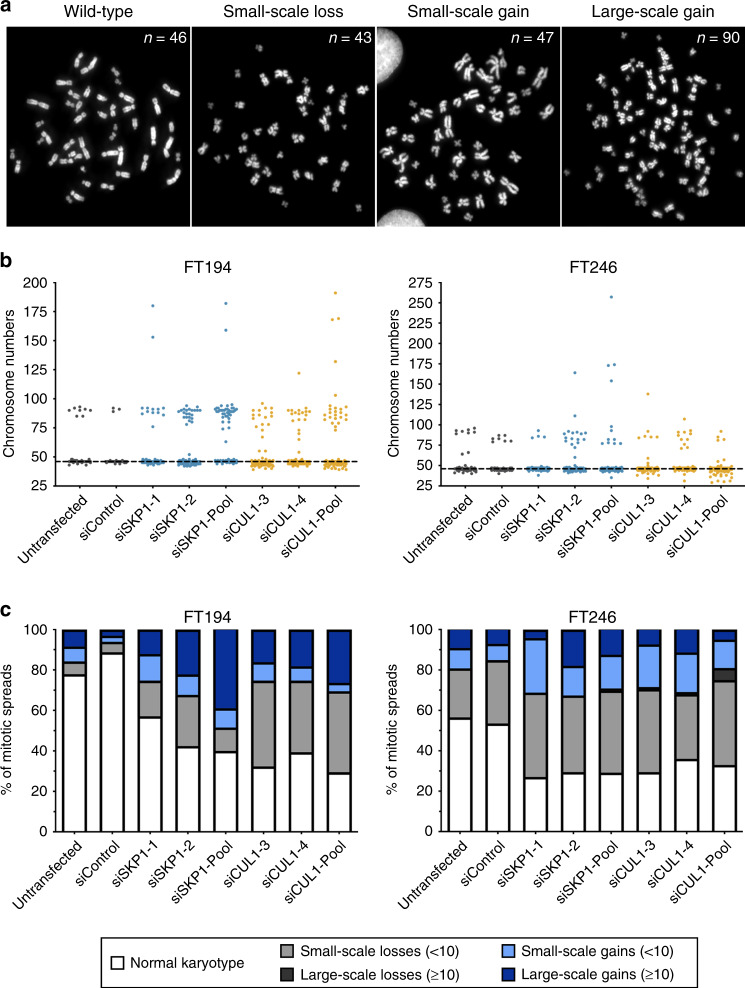
*SKP1* or *CUL1* silencing induces chromosome gains and losses in FT cells. **a** Representative images of mitotic chromosome spreads depicting a normal karyotype and numerical changes including small-scale losses, small-scale gains and large-scale gains. **b** Dot plots depicting chromosome numbers in control cells and following *SKP1* or *CUL1* silencing in FT194 and FT246 cells. The horizontal dotted line identifies the modal number of chromosomes for control cells (*n* = 46). *n* = 2; 100 spreads analysed/condition. **c** Bar charts depicting the frequencies of small-scale (<10) or large-scale (≥10) chromosome gains and losses relative to the modal chromosome number in FT194 and FT246 cells. *n* = 2; 100 spreads analysed/condition.

While the above findings support reduced *SKP1* or *CUL1* expression induces CIN in FT cells, they do not specifically evaluate chromosomes. Thus, to validate these aberrant phenotypes arise due to underlying changes in chromosome numbers, mitotic chromosome spreads were generated, and chromosome numbers were compared between *SKP1* or *CUL1* silenced and siControl conditions. Figure 6a presents representative chromosome spreads obtained from FT194 cells exhibiting the modal karyotype (46 chromosomes) and those exhibiting aberrant numbers of chromosomes. Chromosomes were manually enumerated from 100 mitotic chromosome spreads/condition in both FT194 and FT246 cells and are presented in Fig. 6b. In general, deviations from the modal chromosome number for each line were sub-categorised into small-scale losses or gains involving fewer than ten chromosomes, or large-scale losses or gains, involving ten or more chromosomes (Fig. 6c). Within the FT194 cells, the overall frequencies of aberrant chromosome numbers increased ~4.8-fold for *SKP1* (43–60%) and ~5.9-fold for *CUL1* (61–71%) silencing relative to siControl (11%), with changes including both gains and losses. Large-scale chromosome gains were prevalent, particularly in *SKP1*-silenced cells, while large-scale chromosome losses were never observed. K–S tests determined that the cumulative chromosome number distribution frequencies following *SKP1* or *CUL1* silencing in FT194 cells were statistically distinct from that of the siControl (Supplementary Table SVIII).

As indicated above, the FT246 parental cell line contains two subclones, with subclones 1 and 2 harbouring 46 and 45 chromosomes, respectively. For the purpose of this study, FT246 was considered to have a modal number of 46 chromosomes. Accordingly, the baseline percentage of ‘abnormal’ chromosome spreads within siControl cells is greater within FT246 than FT194, as the ‘small-scale chromosome losses’ class includes subclone 2 cells. In agreement with the FT194 data, *SKP1* or *CUL1* silencing induced overall increases in the proportion of spreads with aberrant chromosome numbers, with a general increase in both small-scale losses and gains—large-scale gains also occurred but were less common. Despite these visual changes, K–S tests (Supplementary Table SIX) failed to identify statistically significant differences in the cumulative distribution frequencies following *SKP1* or *CUL1* silencing relative to siControl; however, K–S tests are particularly insensitive to differences occurring within the tail regions of the distribution curves (i.e., the extreme phenotypes). Nevertheless, the overall percentages of aberrant chromosome numbers in FT246 increased ~1.6-fold following *SKP1* (71–73%) silencing and ~1.5-fold following *CUL1* (64–71%) silencing relative to siControl (46%). Collectively, these findings along with those of the nuclear area and MN formation analyses identify *SKP1* and *CUL1* as novel CIN genes in two independent FT cellular contexts.

### Heterozygous loss of *SKP1* induces dynamic changes in CIN phenotypes in FT246 cells

Heterozygous loss of *SKP1* is the most frequently observed *SKP1* alteration within HGSOC samples (Fig. 1). To model this alteration, CRISPR/Cas9 approaches were used to generate two distinct *SKP1* heterozygous knockout clones in FT246 cells, hereafter referred to as *SKP1*^+/−^ 1 and *SKP1*^+/−^ 2, with DNA sequencing identifying the allele-specific edits (Supplementary Fig. S4A). *SKP1*^+/−^ 1 retained a wild-type *SKP1* allele, while *SKP1*^+/−^ 2 is a compound heterozygote that is predicted to express a mutant SKP1 protein in which two prolines are deleted. Heterozygous loss of *SKP1* was further confirmed by semi-quantitative western blotting (Supplementary Fig. S4B), which showed SKP1 protein levels reduced to 48% (*SKP1*^+/−^ 1) and 23% (*SKP1*^+/−^ 2) of endogenous (NT-Control) levels. Similar to the *SKP1* silencing experiments (Fig. 2a and Supplementary Fig. S3B), heterozygous loss resulted in increased Cyclin E1 expression in both clones (Supplementary Fig. S4B), strongly suggesting that the CRISPR-/Cas9-edited genome affected SCF complex function.

**Fig. 7:**
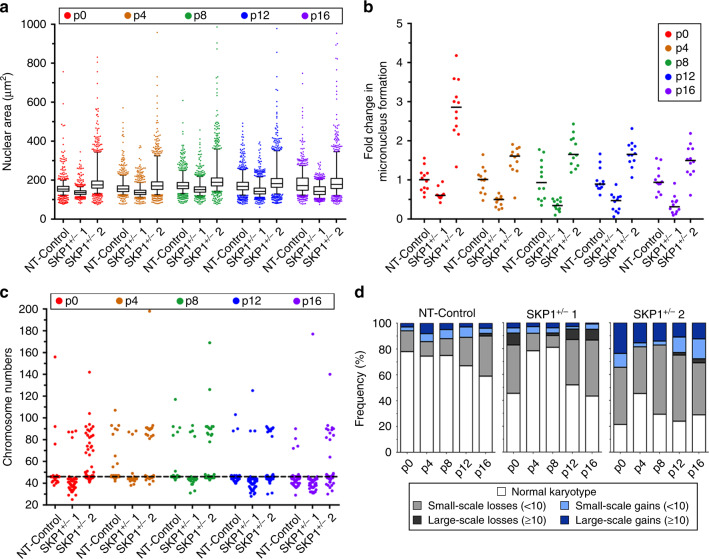
Heterozygous loss of *SKP1* induces dynamic changes in CIN phenotypes and chromosome numbers. **a** Box-and-whisker plots depicting nuclear areas in two distinct *SKP1*^+/−^ clones (*SKP1*^*+/−*^ 1 and 2) and NT-Control at regular time intervals (every four passages (p)) over 2.5 months. Boxes represent the interquartile ranges, with whiskers extending to the 5th and 95th percentiles. *n* = 1; 100 nuclei × 12 replicate wells were analysed/condition/timepoint. **b** Dot plot depicting fold changes in MN formation in *SKP1*^+/−^ clones relative to NT-Control at regular time intervals. Black lines identify the median of 12 replicate wells. *n* = 1; *n* = 12. **c** Dot plot depicting chromosome numbers in *SKP1*^+/−^ clones and NT-Control over time. The horizontal dotted line identifies the modal chromosome number of NT-Control (*n* = 46). *n* = 1; 100 mitotic chromosome spreads were analysed/condition/timepoint. **d** Bar chart depicting the frequencies of small-scale (<10) or large-scale (≥10) chromosome gains and losses relative to the modal chromosome number (*n* = 45 for control and *SKP1*^+/−^ 1; *n* = 46 for *SKP1*^+/−^ 2).

To determine the long-term impact heterozygous *SKP1* loss has on CIN, nuclear area, MN formation and chromosome numbers were assessed in *SKP1*^+/−^ 1, *SKP1*^+/−^ 2 and NT-Control clones at regular time intervals (i.e., every four passages) for ~3 months. Both clones exhibited statistically significant changes in nuclear areas relative to the NT control at each timepoint, with *SKP1*^+/−^ 1 primarily exhibiting decreases in nuclear areas and *SKP1*^+/−^ 2 exhibiting increases in nuclear areas and nuclear area heterogeneity (Fig. 7a), which is consistent with the heterogeneous CIN phenotype (Supplementary Table SX). In addition, *SKP1*^+/−^ 2 (but not *SKP1*^+/−^ 1) exhibited statistically significant increases in MN formation relative to NT control at each timepoint (Fig. 7b and Supplementary Table SXI). Of the two clones evaluated, the nuclear area and MN formation changes observed within *SKP1*^+/−^ 2 most closely resembled those observed following *SKP1* silencing, and exhibited the cell-to-cell heterogeneity that accompanies CIN. However, it is important to note that the nuclear area assay is most sensitive to large-scale changes in chromosome gains (i.e., ploidy) and therefore may not readily identify numerical changes involving only a small subset of chromosomes. Similarly, the MN formation assay is an indicator of small-scale chromosome missegregation events and may not identify all instances of chromosome gains or losses.

To validate the nuclear area and MN formation findings and gain additional insight into the evolving chromosome complements contained within the *SKP1*^+/−^ clones, mitotic chromosome spreads were analysed at each timepoint. As shown in Fig. 7c, chromosome numbers were dynamic in both *SKP1*^+/−^ clones and fluctuated between time points, which is reflected by statistically different cumulative distribution frequencies relative to the corresponding NT-Control at each time point (Supplementary Table SXII). It is important to note that the NT-Control and *SKP1*^+/−^ 2 clones have a modal chromosome number of 46, while the *SKP1*^+/−^ 1 clone has a modal chromosome number of 45, indicating that the initial clones were derived from the two distinct subclones pre-existing within the parental FT246 cell line. Accordingly, changes in chromosome numbers were categorised as small-scale (<10) or large-scale (≥10) gains or losses relative to the modal chromosome number specific to each clone (Fig. 7d). In agreement with the silencing data, heterozygous loss of *SKP1* corresponded with an overall increase in the frequency of spreads with aberrant chromosome numbers. Interestingly, *SKP1*^+/−^ 1 was most susceptible to chromosome losses (small-scale and large-scale) that were most evident at p0, p12 and p16, while *SKP1*^+/−^ 2 exhibited chromosome losses (small-scale) and gains (small-scale and large-scale) that were present at all five time points. Overall, the frequencies of abnormal chromosome numbers ranged from ~20 to 55% for *SKP1*^+/−^ 1 and ~55–80% for *SKP1*^+/−^ 2, and are consistent with *SKP1*^+/−^ 2 having a higher level of CIN compared with *SKP1*^+/−^ 1. This difference may be due to their different genetic backgrounds, the fact that *SKP1*^+/−^ 2 is a compound heterozygote with structural changes that may affect the normal function of the expressed protein or a difference in the evolutionary trajectory of these populations during clonal expansion. Nevertheless, analysis of mitotic chromosome spreads revealed ongoing cell-to-cell heterogeneity and CIN within both *SKP1*^+/−^ clones.

## Discussion

Loss of chromosome stability is consistent with early steps leading to cancer formation. Consistent with the loss of chromosome stability being an early event in HGSOC, TCGA data show that both *SKP1* and *CUL1* exhibit shallow deletions (i.e., heterozygous loss) in HGSOC patient samples. To identify the potential mechanism by which reduced *SKP1* or *CUL1* expression may contribute to early disease development, we employed two complementary approaches. First, transient siRNA-based silencing of *SKP1* or *CUL1* was shown to induce increases in Cyclin E1 levels and CIN phenotypes within two distinct FT cell lines. Importantly, this work provided critical functional insight into the relationship between SKP1 or CUL1 and their modulation of Cyclin E1 levels and chromosome stability and identified both *SKP1* and *CUL1* as novel CIN genes within HGSOC precursor cells. Second, two *SKP1*^+/−^ cell lines were shown to develop ongoing changes in chromosome numbers, increased Cyclin E1 levels and distinct CIN phenotypes. Overall, this work provides a foundation on which to model the early aetiologic events contributing to HGSOC pathogenesis.

While each of the approaches employed to reduce target gene expression reproducibly induced increases in CIN phenotypes, the type and severity of CIN phenotypes differed between approaches. It is important to note that CIN is a heterogeneous phenotype, and different chromosome complements may confer distinct survival advantages or disadvantages leading to the divergence of karyotypic patterns observed between different samples/conditions. This is especially true for the heterozygous knockout approach, where stable (CRISPR/Cas9) reduction in SKP1 expression and long-term culturing of cells requires cells remain viable through multiple rounds of mitosis and that cell proliferation rates are not adversely impacted. It is likely that cells with ‘extreme’ levels of CIN are not viable and are lost from the population. Thus, the phenotypic differences observed between the two *SKP1*^+/−^ subclones begin to provide novel insight into how CIN-positive cells may adapt and evolve over long-term growth, and thus provides novel insight into the extensive tumour cell and genomic heterogeneity observed in HGSOC.

Another unexpected finding was that while both *SKP1* and *CUL1* silencing induced increases in CIN phenotypes, the phenotypes were more pronounced within the *SKP1*-silenced cells. While it is possible that these variances may stem from technical factors such as differences in gene silencing efficiencies, semi-quantitative western blot analyses following *SKP1* or *CUL1* silencing consistently show a comparable and strong reduction in both SKP1 and CUL1 levels. Thus, these data suggest that there are functional differences between SKP1 and/or CUL1 even though they are both components of the SCF complex. Emerging evidence from several recent studies has begun to show that SKP1 interacts with additional binding partners beyond the SCF complex,^71–75^ which may potentially exacerbate the CIN phenotypes associated with reduced *SKP1* expression. In addition, cullin 3 (CUL3) was recently identified as an important mediator of Cyclin E degradation via the ubiquitin–proteasome pathway,^76^ and may contribute to the less striking phenotypes observed with *CUL1* knockdown. Our data showing a partial rescue of the nuclear area and MN phenotypes with *CCNE1* co-silencing further implicate other downstream targets of the SCF complex contributing to the CIN phenotype in FT cells. Overall, further studies investigating the functional differences between SKP1 and CUL1, including potential compensatory pathways are now warranted.

Several studies have previously induced transformation of immortalised FT cells via overexpression of various oncogenes,^7,48,64^ but the role CIN plays in FT cell transformation has never been evaluated. For example, Karst et al.^21^ demonstrated that overexpression of Cyclin E1 within FT282 cells induced cellular transformation-associated phenotypes including increases in cell proliferation rates, clonogenic growth, loss of contact inhibition and mild increases in anchorage-independent growth (i.e., soft agar colony formation). The current study builds upon that model by showing that reduced degradation of Cyclin E1 stemming from a diminished expression of SCF complex components, namely SKP1 and CUL1, is an additional mechanism by which Cyclin E1 levels may increase in cancer cells. Indeed, while Cyclin E1 protein levels are elevated in ~45–50% of HGSOC cases,^30,31^ genomic amplification of the Cyclin E1 (*CCNE1*) gene only occurs in ~20% of cases.^29^ This study identifies a novel mechanism whereby diminished SKP1 or CUL1 expression, and thus reduced SCF complex activity, may lead to increased Cyclin E1 protein levels that correspond with increases in CIN. Furthermore, heterozygous loss of *SKP1* in FT246 cells induced increases in Cyclin E1 and CIN phenotypes that were sustained within the population over an extended period of time. Collectively, these fundamental findings implicate diminished SCF function as a potential pathogenic event in HGSOC, and may account for a large proportion of the ~20–25% of HGSOC cases exhibiting increased Cyclin E1 levels, but for which *CCNE1* is not genomically amplified. The results shown herein represent an early step towards uncovering the role of CIN in HGSOC pathogenesis. Finally, the results of this study may have far-reaching implications beyond HGSOC, as heterozygous loss of *SKP1* or *CUL1* occurs in at least 12 common cancer types, although this remains to be empirically determined.

## Supplementary information

Supplemental Tables I-XII and Figures S1-S4

## Data Availability

Patient-related data (Fig. 1) are based on the data generated by the TCGA Research Network and are available at https://www.cancer.gov/tcga. All descriptive statistics and statistical analyses presented in Figs. 3–7 are provided within Supplementary Tables SII–SXII.
