# Supplementary material for: Reduced SKP1 and CUL1 expression underlies increases in Cyclin E1 and chromosome instability in cellular precursors of high-grade serous ovarian cancer
Source: Br J Cancer. 2021 Mar 17;124(10):1699–710. doi: 10.1038/s41416-021-01317-w (PMC8110794; doi:10.1038/s41416-021-01317-w)
Supplement: Supplementary file 1 — Supplemental Tables I-XII and Figures S1-S4 [file 41416_2021_1317_MOESM1_ESM.pdf]

SUPPLEMENTARY INFORMATION

SUPPLEMENTARY TABLES

Table S I. List of Antibodies Employed for Western Blot Analyses.

Target Protein[A]	Species	Company	Catalog Number	Working Dilution
α-Tubulin	Mouse	Abcam	ab7291	1:20,000
CCNE1	Rabbit	Abcam	ab33911	1:1,000
CUL1	Rabbit	Abcam	ab75812	1:1,000
Cyclophilin B	Rabbit	Abcam	ab16045	1:50,000
SKP1	Rabbit	Abcam	ab76502	1:2,000
Anti-Rabbit HRP	Goat	Jackson ImmunoResearch	111-035-144	1:15,000
Anti-Mouse HRP	Goat	Jackson ImmunoResearch	115-035-146	1:10,000

[A]HRP (Horseradish peroxidase)

Table S II. K-S Tests Identify Significant Changes in Nuclear Area Cumulative Distribution Frequencies Following *SKP1* Silencing in FT194 Cells.

Condition	n[A]	*p*-value[B]	Significance[C]	D-statistic[D]
Untransfected	300	0.0812	ns	0.1033
siControl	300	N/A	N/A	N/A
siSKP1-1	300	< 0.0001	****	0.2633
siSKP1-2	300	< 0.0001	****	0.2600
siSKP1-Pool	300	< 0.0001	****	0.2067
siCUL1-3	300	< 0.0001	****	0.2600
siCUL1-4	300	0.0209	*	0.1233
siCUL1-Pool	300	0.2099	ns	0.0867

[A]Number of nuclei analyzed.
[B]*p*-values calculated from two-sample K-S tests for the listed condition relative to siControl. N/A, not applicable.
[C]Significance (ns, not significant; *, *p*-value < 0.05; ****, *p*-value < 0.0001).
[D]D-statistic (maximum deviation between the two distribution curves).

Table S III. M-W Tests Identify Significant Increases in MN Formation Following *SKP1* or *CUL1* Silencing in FT194 Cells.

Condition	n[A]	Mean Nucleus Count[B]	Mean MN Count[C]	Mean MN Formation (%)[D]	Fold Change in MN Formation[E]	*p*-value[F]
Untransfected	6	1605	169	10.57	1.07	0.3095
siControl	6	1583	153	9.61	0.97	N/A
siSKP1-1	6	1004	182	18.80	1.89	0.0022
siSKP1-2	6	1147	218	20.70	1.80	0.0022
siSKP1-Pool	6	885	154	18.65	1.86	0.0022
siCUL1-3	6	1280	197	15.60	1.58	0.0022
siCUL1-4	6	1518	151	10.00	1.03	0.2403
siCUL1-Pool	6	1507	203	13.48	1.30	0.0087

[A]Number of wells analyzed.
[B]Mean number of nuclei analyzed per well.
[C]Mean number of micronuclei counted per well.
[D]Mean percent MN formation (calculated for each well as the MN count/nucleus count × 100).
[E]Median fold change in MN formation relative to siControl at the corresponding timepoint.
[F]*p*-values calculated from two-sample M-W tests for the listed condition relative to siControl at the corresponding timepoint. N/A, not applicable.

Table S IV. K-S Tests Identify Significant Changes in Nuclear Area Cumulative Frequency Distributions Following *SKP1* Silencing in FT246 Cells.

Condition	n[A]	*p*-value[B]	Significance[C]	D-statistic[D]
Untransfected	300	0.2485	ns	0.0833
siControl	300	N/A	N/A	N/A
siSKP1-1	300	< 0.0001	****	0.3767
siSKP1-2	300	< 0.0001	****	0.2414
siSKP1-Pool	300	< 0.0001	****	0.3833
siCUL1-3	300	0.0005	***	0.1667
siCUL1-4	300	0.0659	ns	0.2076
siCUL1-Pool	300	0.0002	***	0.1733

[A]Number of nuclei analyzed.
[B]*p*-values calculated from two-sample K-S tests for the listed condition relative to siControl. N/A, not applicable.
[C]Significance (ns, not significant; ***, p-value < 0.001; ****, p-value < 0.0001).
[D]D-statistic (maximum deviation between the two distribution curves).

S V. M-W Tests Identify Significant Increases in MN Formation Following *SKP1* or *CUL1* Silencing in FT246 Cells.

**Condition**	**n[A]**	**Mean Nucleus Count[B]**	**Mean MN Count[C]**	**Mean MN Formation (%)[D]**	**Fold Change in MN Formation[E]**	***p*-value[F]**
Untransfected	6	516	11	2.11	0.93	0.8182
siControl	6	604	14	2.28	0.99	N/A
siSKP1-1	6	165	18	10.64	4.08	0.0022
siSKP1-2	6	268	21	7.96	3.94	0.0260
siSKP1-Pool	6	180	16	8.45	3.98	0.0022
siCUL1-3	6	436	31	7.10	3.05	0.0022
siCUL1-4	6	557	18	3.26	1.30	0.1797
siCUL1-Pool	6	538	33	6.09	2.77	0.0043

[A]Number of wells analyzed.
[B]Mean number of nuclei analyzed per well.
[C]Mean number of micronuclei counted per well.
[D]Mean percent MN formation (calculated for each well as the MN count/nucleus count × 100).
[E]Median fold change in MN formation relative to siControl at the corresponding timepoint.
[F]*p*-values calculated from two-sample M-W tests for the listed condition relative to siControl at the corresponding timepoint. N/A, not applicable.

Table S VI: Co-silencing *SKP1* and *CCNE1* reduces micronucleus formation[A].

	**siControl**	**siCCNE1**	**siSKP1**	**siSKP1 + siCCNE1**
**Untransfected**	0.9989	0.9907	0.0031	0.6147
**siControl**		0.9518	0.0016	0.4585
**siCCNE1**			0.0093	0.8631
**siSKP1**				0.0842

[A]Tukey multi-comparison post-tests

Table S VII: Co-silencing *CUL1* and *CCNE1* reduces micronucleus formation[A].

	**siControl**	**siCCNE1**	**siCUL1**	**siCUL1 + siCCNE1**
**Untransfected**	0.9993	0.9939	<0.0001	0.0009
**siControl**		0.9673	<0.0001	0.0005
**siCCNE1**			0.0001	0.0026
**siCUL1**				0.7609

[A]Tukey multi-comparison post-tests

Table S VIII. K-S Tests Identify Significant Changes in Cumulative Chromosome Number Frequency Distributions Following *SKP1* or *CUL1* Silencing in FT194 Cells.

Condition	n[A]	*p*-value[B]	Significance[C]	D-statistic[D]
Untransfected	100	0.7736	ns	0.0900
siControl	100	N/A	N/A	N/A
siSKP1-1	100	0.0327	*	0.2076
siSKP1-2	100	0.0023	**	0.2620
siSKP1-Pool	100	< 0.0001	****	0.4284
siCUL1-3	100	< 0.0001	****	0.1417
siCUL1-4	100	0.0002	***	0.3090
siCUL1-Pool	100	< 0.0001	****	0.3552

[A]Number of mitotic spreads analyzed.

[B]*p*-values calculated from two-sample K-S tests for the listed condition vs. siControl. N/A, not applicable.

[C]Significance (ns, not significant; *, *p*-value < 0.05; **, *p*-value < 0.01; ***, *p*-value < 0.001; ****, *p*-value < 0.0001).

[D]D-statistic (maximum deviation between the two distribution curves).

Table S IX. Statistical Assessment of Chromosome Number Distributions Following *SKP1* or *CUL1* Silencing in FT246 Cells.

Condition	n[A]	*p*-value[B]	Significance[C]	D-statistic[D]
Untransfected	100	0.8127	ns	0.0900
siControl	100	N/A	N/A	N/A
siSKP1-1	100	0.1644	ns	0.1593
siSKP1-2	100	0.1069	ns	0.1729
siSKP1-Pool	100	0.3001	ns	0.1387
siCUL1-3	100	0.2793	ns	0.1417
siCUL1-4	100	0.1790	ns	0.1561
siCUL1-Pool	100	0.0171	*	0.2176

[A]Number of mitotic spreads analyzed.

[B]*p*-values calculated from two-sample K-S tests for the listed condition vs. siControl. N/A, not applicable.

[C]Significance (ns, not significant; *, p-value < 0.05).

[D]D-statistic (maximum deviation between the two distribution curves).

Table S X. Statistical Assessment of Nuclear Area Cumulative Distribution Frequencies in *SKP1*$^{+/-}$ FT246 Clones Over Time.

Passage	Clone	n[A]	*p*-value[B]	Significance[C]	D-statistic[D]
p0	Control	1200	N/A	N/A	N/A
	SKP1$^{+/-}$ 1	1200	< 0.0001	****	0.4467
	SKP1$^{+/-}$ 2	1200	< 0.0001	****	0.3850
p4	Control	1200	N/A	N/A	N/A
	SKP1$^{+/-}$ 1	1200	< 0.0001	****	0.4025
	SKP1$^{+/-}$ 2	1200	< 0.0001	****	0.2650
p8	Control	1200	N/A	N/A	N/A
	SKP1$^{+/-}$ 1	1200	< 0.0001	****	0.3667
	SKP1$^{+/-}$ 2	1200	< 0.0001	****	0.2725
p12	Control	1200	N/A	N/A	N/A
	SKP1$^{+/-}$ 1	1200	< 0.0001	****	0.3933
	SKP1$^{+/-}$ 2	1200	< 0.0001	****	0.2050
p16	Control	1200	N/A	N/A	N/A
	SKP1$^{+/-}$ 1	1200	< 0.0001	****	0.3525
	SKP1$^{+/-}$ 2	1200	< 0.0001	****	0.1050

[A]Number of nuclei analyzed.
[B]*p*-values calculated from two-sample K-S tests for the listed condition relative to siControl at the corresponding timepoint. N/A, not applicable.
[C]Significance (****, *p*-value < 0.0001).
[D]D-statistic (maximum deviation between the two distribution curves).

Table S XI. Statistical Assessment of MN Formation in *SKP1*$^{+/-}$ FT246 Clones Over Time.

Passage	Clone	n[A]	Mean Nucleus Count[B]	Mean MN Count[C]	Mean MN Formation (%)[D]	Fold Change in MN Formation[E]	*p*-value[F]
p0	Control	12	737	22	2.95	1.00	N/A
	SKP1$^{+/-}$ 1	12	1124	21	1.85	0.60	0.0007
	SKP1$^{+/-}$ 2	12	503	42	8.34	2.86	< 0.0001
p4	Control	12	405	14	3.51	1.01	N/A
	SKP1$^{+/-}$ 1	12	731	12	1.58	0.50	< 0.0001
	SKP1$^{+/-}$ 2	12	498	26	5.26	1.61	0.0014
p8	Control	12	617	26	4.30	0.93	N/A
	SKP1$^{+/-}$ 1	12	515	8	1.49	0.34	0.0002
	SKP1$^{+/-}$ 2	12	597	44	7.44	1.65	0.0023
p12	Control	12	618	29	4.72	0.89	N/A
	SKP1$^{+/-}$ 1	12	438	9	1.99	0.47	< 0.0001
	SKP1$^{+/-}$ 2	12	506	40	7.89	1.65	< 0.0001
p16	Control	12	428	17	3.97	0.94	N/A
	SKP1$^{+/-}$ 1	12	517	7	1.42	0.31	< 0.0001
	SKP1$^{+/-}$ 2	12	478	28	5.89	1.50	0.0068

[A]Number of wells analyzed.
[B]Mean number of nuclei analyzed per well.
[C]Mean number of micronuclei enumerated per well.
[D]Mean percent MN formation (calculated for each well as the MN count/Nucleus count × 100).
[E]Median fold change in MN formation relative to siControl at the corresponding timepoint.
[F]*p*-values calculated from two-sample M-W tests for the listed condition relative to siControl at the corresponding timepoint. N/A, not applicable.

Table S XII. Statistically Significant Changes in Cumulative Chromosome Number Frequency Distributions in *SKP1*$^{+/-}$ FT246 Clones Over Time.

Passage	Clone	n[A]	*p*-value[B]	Significance[C]	D-statistic[D]
p0	Control	100	N/A	N/A	N/A
	SKP1$^{+/-}$ 1	100	< 0.0001	****	0.8292
	SKP1$^{+/-}$ 2	100	0.0008	***	0.2841
p4	Control	100	N/A	N/A	N/A
	SKP1$^{+/-}$ 1	100	< 0.0001	****	0.8869
	SKP1$^{+/-}$ 2	100	0.0048	**	0.2486
p8	Control	100	N/A	N/A	N/A
	SKP1$^{+/-}$ 1	100	< 0.0001	****	0.8900
	SKP1$^{+/-}$ 2	100	< 0.0001	****	0.4054
p12	Control	100	N/A	N/A	N/A
	SKP1$^{+/-}$ 1	100	< 0.0001	****	0.7800
	SKP1$^{+/-}$ 2	100	0.0001	***	0.3100
p16	Control	100	N/A	N/A	N/A
	SKP1$^{+/-}$ 1	100	< 0.0001	****	0.6785
	SKP1$^{+/-}$ 2	100	0.0415	*	0.1984

[A]Number of mitotic spreads analyzed.
[B]*p*-values calculated from two-sample K-S tests for the listed condition relative to siControl at the corresponding timepoint. N/A, not applicable.
[C]Significance (*, *p*-value < 0.05; **, *p*-value < 0.01; ***, *p*-value < 0.001; ****, *p*-value < 0.0001).
[D]D-statistic (maximum deviation between the two distribution curves).

A

FT194

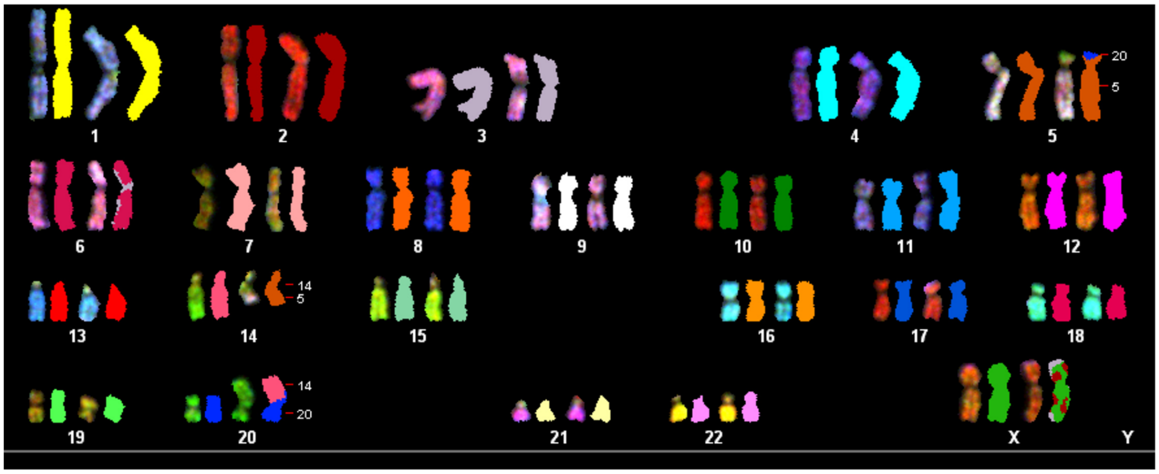

B

FT246 - Subclone 1

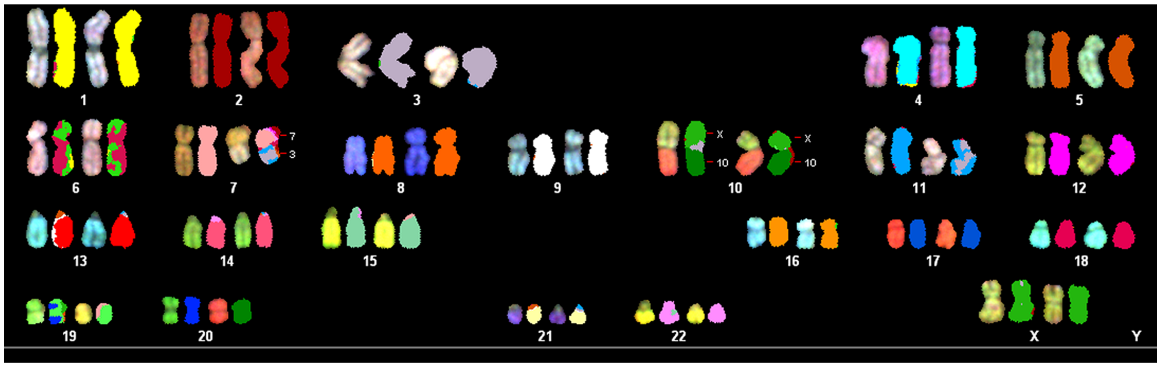

FT246 - Subclone 2

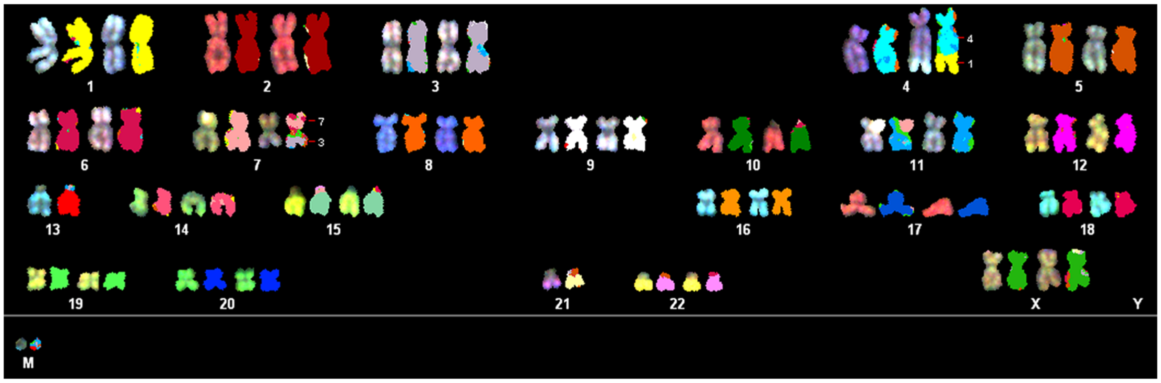

Figure S1. Spectral Karyotyping of FT194 and FT246 Cells.
(A) The modal spectral karyotype for FT194 is 46,XX,t(5;14;20)(p1?5;q2?4;p1?3) based on the analysis of 25 karyotypes. **(B)** Based on the analysis of 25 karyotypes, FT246 harbor two predominant karyotypes 46,XX,der(7)t(3;7),-10,-10,+der(10)t(10;X)x2,del(X)(q?) (subclone 1; top), and 45,XX,der(4)t(1;4),der(7)t(3;7),del(10)(p11.2),-13,-21,+mar (subclone 2; bottom).

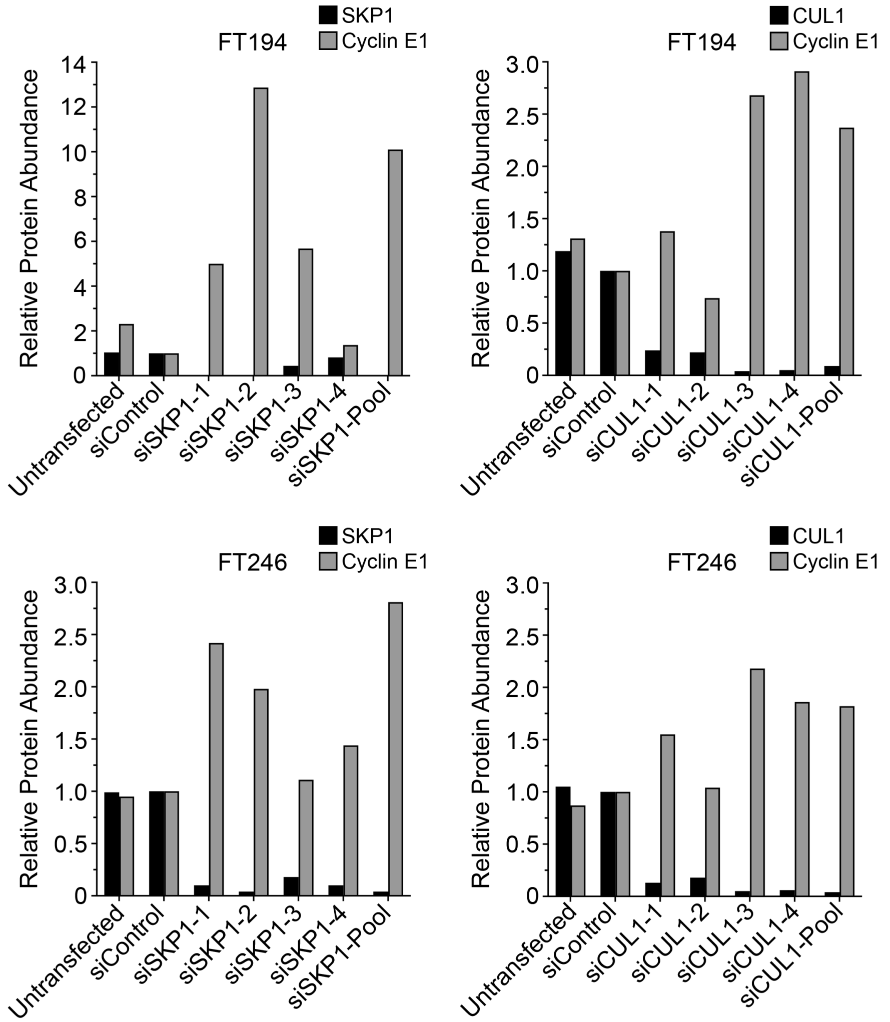

Figure S2. ***SKP1*** **or** ***CUL1*** **Silencing is Associated with Increases in Cyclin E1 Abundance.** Bar charts of relative protein abundance representing the semi-quantitative western blots depicting the *SKP1* or *CUL1* silencing efficiency as shown in Figure 2.

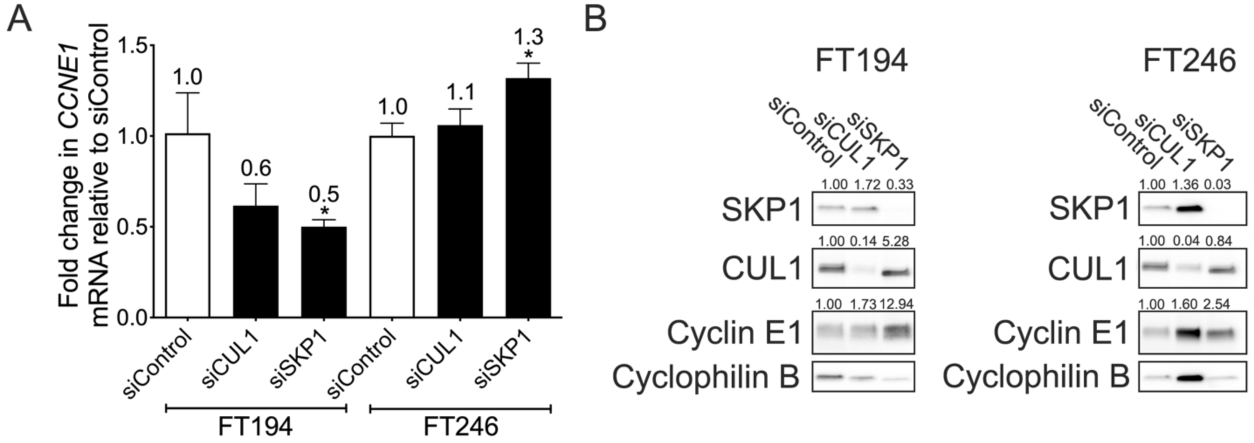

Figure S3. Evaluation of *CCNE1* transcripts and Cyclin E1 protein after *SKP1* or *CUL1* knockdown. (A) Mean *CCNE1* RNA expression data from FT194 and FT246 cells after transfection with siControl, siSKP1 or siCUL1. Data are shown as fold change in expression relative to siControl. Bars represent standard deviation from three independently transfect plates. (*, *P*-value < 0.05). **(B)** Semi-quantitative western blot depicting the silencing efficiencies of individual siRNA treatments with the numerical values indicating residual protein levels. Fold changes in SKP1, CUL1 or Cyclin E1 expression relative to siControl are indicated, using cyclophilin B as a loading control.

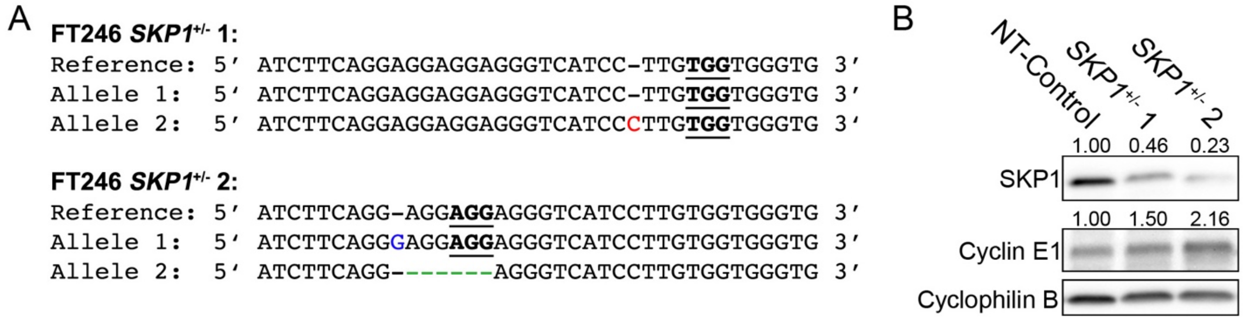

Figure S4. DNA Sequencing and Western Blots Identify CRISPR/CAS9 Edits of *SKP1*.
(A) DNA sequencing results for individual *SKP1* alleles showing small indels (colored text) at the expected CRISPR edit site located 3 base pairs (bp) upstream of the NGG recognition sequence (protospacer adjacent motif [PAM]; underlined). *SKP1*$^{+/-}$ 1 retains one wild-type allele and harbors a 1 bp (out-of-frame) insertion in the second allele. *SKP1*$^{+/-}$ 2 harbors a 1 bp (out-of-frame) insertion in one allele, and a 6 bp (in-frame) deletion in the second allele. **(B)** Representative semi-quantitative western blots depicting the SKP1 and Cyclin E1 expression in FT246 *SKP1* heterozygous clones (*SKP1*$^{+/-}$1 and *SKP1*$^{+/-}$2). Fold changes in SKP1 or Cyclin E1 expression relative to NT-Control are indicated, using cyclophilin B as a loading control.
